# Tick-borne Encephalitis in Southern Norway

**DOI:** 10.3201/eid1003.020734

**Published:** 2004-03

**Authors:** Péter A. Csángó, Ellef Blakstad, Georges C. Kirtz, Judith E. Pedersen, Brigitte Czettel

**Affiliations:** * Vest-Agder Hospital, Kristiansand, Norway; † Norwegian Veterinary Association, Oslo, Norway; ‡ InVitro, Labor für Veterinärmedizinische Diagnostik und Hygiene GmbH, Vienna, Austria

**Keywords:** tick-borne encephalitis, antibodies, incidence, dogs, Norway

## Abstract

The first five cases of human tick-borne encephalitis in Norway were reported from Tromöya, in Aust-Agder County. Serum specimens from 317 dogs in the same geographic area were collected. An enzyme immunoassay demonstrated antibody to human tick-borne encephalitis virus in 52 (16.4%) of the dogs, which supports the notion of an emerging disease.

First described in humans in Austria (*1*), tick-borne encephalitis (TBE) is rapidly becoming a growing public health problem in Europe (*2*). Although observations indicated antibody presence in humans in southern Norway (*3*), this country has been absent from maps visualizing TBE-endemic areas. This situation may be changing. The first case of clinically manifest TBE in humans in Norway was reported in 1998 (*4*). Four additional cases were described from 1998 to 2001; all five cases were from Tromøya in Aust-Agder County of southern Norway (*5*). Infected dogs indicate that TBE virus (TBEV) is present in different geographic areas. The first case of TBE in dogs was reported by Lindblad in Sweden (*6*), and later by others in Central Europe (*7*). We investigated and found TBEV immunoglobulin (Ig) G in dogs in southern Norway, an area where this virus was not previously considered endemic.

## The Study

From 1992 to 2000, we collected serum samples from 317 (65 breeds, 146 male, 171 female) dogs seen at a veterinary clinic in Arendal, in southern Norway. The laboratory received 436 serum specimens. In case of multiple specimens from one dog, collected during several months or years, we controlled the results for possible changes in antibody levels.

We used two different enzyme-linked immunosorbent assay (ELISA) techniques. The presence and level of IgG antibodies to TBEV were tested by an enzyme immunoassay for the detection of IgG antibodies to TBEV (Enzygnost Anti-TBE virus IgG, Dade Behring Marburg GmbH, Marburg, Germany). Antibody levels >1:100 were considered positive. Controls were obtained from the laboratory InVitro (InVitro, Vienna, Austria). IgG to TBEV was detected by a specific sheep, anti-dog, heavy and light chain IgG antibody (A40-105P-7, Bethyl Laboratories, Montgomery, TX) in a dilution of 1:20,000. Positive specimens were confirmed by a second ELISA (Baxter-Immuno, Orth, Austria), as previously described (*7*). In this assay, titers >100 were considered to be positive.

## Results

A total of 52 (16.4%) of 317 dogs had IgG antibodies to TBEV; 40 (12.6%) had IgG antibody titers to TBEV >450, while 12 dogs (3.8%) had moderate levels (>100–<450) (Table 1). Positive serum specimens, including samples with 11 to <100 U in the enzymeimmunoassay (EIA)-E test, were confirmed with the Baxter-Immuno (B-I) test (Table 2). The confirmatory test included five extra serum samples in instances where such blood samples were drawn; thus the number of positive specimens to be confirmed was 57.

**Table 1:** Serologic examination of canine serum specimens for antibodies to tick-borne encephalitis virus

U	N
<100	265
>100	52
Total	317

**Table 2:** Distribution of positive canine serum specimens^a^

EIA-E U >450	N	EIA-E U >100–<450	N	EIA-E U >11–<100	N
B-I titer		B-I titer		B-I titer	
3,200	1	3,200	0	3,200	0
1,600	5	1,600	0	1,600	0
800	5	800	0	800	0
400	18	400	4	400	0
200	12	200	2	200	1
100	3	100	3	100	3
Total	44		9		4

^a^Positive by enzyme immunoassay E-test (EIA-E) and confirmed by a second test (enzyme immunoassay Baxter-Immuno [EIA-BI]). In five cases, we had two or more serum specimens with high positive results. All these samples were tested by both enzyme-linked immunosorbent assay techniques.

We could not confirm one result (no. 287) with 116 U in the Enzygnost (EIA-E) by the Immuno ELISA. Of the low-positive specimens in the Enzygnost (<100 U), only four specimens had low-positive results in the B-I ELISA; all others were negative. Four low-positive EIA-E specimens gave positive results in the B-I test. On the other hand, 9 low-positive specimens in the EIA-E (20–37 U) were negative by the B-I test.

The codes were not broken until after the experiments were performed. Thus serum specimens sampled and coded at different times were in some cases collected from a single dog. Nevertheless, high positive antibody levels were reproducible even after several years. In five instances, we had two or more serum specimens from one dog with high positive results at our disposal. All these samples were tested by both ELISA techniques.

Only results of >450 U in the Enzygnost test could be registered, which in two instances gave lower results in the new specimens. The Immuno ELISA was in agreement with the Enzygnost in case A, and it showed stable titers in case B. In cases C and D, one could observe an increase in titers by the B-I test. We observed seroconversion in three cases.

The average age of the dogs at the time of blood sampling was 6.6 years (0.5–15). The 52 dogs with ≥100 U were 8.02 years versus dogs with <100 U, which were younger, 6.29 years. The distribution of antibodies according to the size of the dogs is shown in Table 3. A total of 34 (21.8%) of 151 large dogs had antibodies to TBEV ≥100 U versus 18 (10.8%) of 166 small and medium-sized dogs. Large dogs were defined as having a body weight of >20 kg. This difference is statistically significant: with odds ratio = 2.39, χ^2^ = 7.03, p = 0.008 with Yates’ correction. Among dogs with >450 U, 25 (62.5%) of 40 were large.

**Table 3:** Distribution of antibodies according to size of dog

Breed	N positive serum specimens >100 U	N negative serum specimens <100 U	Total	% positive
Alsatian wolf dog	7	33	40	21.2
Bernese Mountain dog	1	7	8	14.3
Bouvier	1	6	7	16.7
Finnish dog	1	4	5	25.0
Flat Coated Retriever	5	11	16	45.4
Golden Retriever	4	21	25	19.0
Hovawart	1	2	3	33.3
Labrador Retriever	5	16	21	31.2
Newfoundland dog	1	8	9	12.5
Giant Schnauzer	3	3	6	50.0
Rottweiler	2	7	9	28.6
St. Bernard	3	0	3	100.0
Total, large dogs	34	117	151	22.5
Total, other dogs	18	148	166	10.8
Total, all dogs	52	265	317	16.4

## Conclusions

Antibodies to TBEV were detected in 16.4% of dogs in Aust-Agder County of southern Norway. This finding indicates that TBEV is present in this geographic region. Although the first human cases prove the existence of TBEV in southern Norway, the levels of seropositivity in dogs were still unanticipated in a region where TBE has previously not been seen.

TBE in dogs has been reported from several European countries (*7*), and the number of cases is growing. Searching for antibodies to TBEV in our canine population would be useful since dogs are suitable serologic indicators of TBEV in a geographic area, and canine serum has been used to reveal natural epidemic foci. Our data support the recent findings of human TBE cases in Norway and the notion of an emerging disease, especially because the serum samples were collected from the same geographic area where the first human cases were described. The changing epidemiologic situation suggests that better monitoring of TBE is needed in Norway.

## References

[1] Schneider H. On the epidemic acute serous meningitis. [German.]. Wien Klin Wochenschr. 1931;44:350–2.

[2] Haglund M. Occurrence of TBE in areas previously considered being non-endemic. Int J Med Microbiol. 2002;291(Suppl 33):50–4. 10.1016/S1438-4221(02)80010-8 12141758

[3] Skarpaas T, Csángó PA, Pedersen J. Tick-borne encephalitis in southern Norway. MSIS-rapport 2000; No. 9. Oslo: The National Institute of Public Health; 2000. Norwegian.

[4] Ormaasen V, Brantsæter AB, Moen EW. Tick-borne encephalitis in Norway. [Norwegian.]. Tidsskr Nor Laegeforen. 2001;121:807–9. 11301704

[5] Skarpaas T, Sundøy A, Vene S, Pedersen J, Eng PG, Csángó PA. Tick-borne encephalitis in Norway. [Norwegian.]. Tidsskr Nor Laegeforen. 2002;122:30–2. 11851291

[6] Lindblad G. A case of tick-borne encephalitis in a dog. Medlemsblad för Sveriges veterinärforbund 1960;12:416–7. Swedish.

[7] Kirtz G. Tick-borne encephalitis in Austrian dogs [Doctoral dissertation]. Vienna: University of Vienna; 1999.

